# Discovering the pharmacodynamics of conolidine and cannabidiol using a cultured neuronal network based workflow

**DOI:** 10.1038/s41598-018-37138-w

**Published:** 2019-01-15

**Authors:** G. D. C. Mendis, G. Berecki, E. Morrisroe, S. Pachernegg, M. Li, M. Varney, P. B. Osborne, C. A. Reid, S. Halgamuge, S. Petrou

**Affiliations:** 1 Department of Mechanical Engineering, University of Melbourne, Parkville, VIC 3010 Australia; 2 Florey Institute of Neuroscience and Mental Health, University of Melbourne, Parkville, VIC 3010 Australia; 3 Neurolixis, Inc, Dana Point, CA 92629 USA; 4 Department of Anatomy and Neuroscience, University of Melbourne, Parkville, VIC 3010 Australia; 5 Research School of Engineering, College of Engineering and Computer Science, Australian National University, Canberra, ACT 0200 Australia

## Abstract

Determining the mechanism of action (MOA) of novel or naturally occurring compounds mostly relies on assays tailored for individual target proteins. Here we explore an alternative approach based on pattern matching response profiles obtained using cultured neuronal networks. Conolidine and cannabidiol are plant-derivatives with known antinociceptive activity but unknown MOA. Application of conolidine/cannabidiol to cultured neuronal networks altered network firing in a highly reproducible manner and created similar impact on network properties suggesting engagement with a common biological target. We used principal component analysis (PCA) and multi-dimensional scaling (MDS) to compare network activity profiles of conolidine/cannabidiol to a series of well-studied compounds with known MOA. Network activity profiles evoked by conolidine and cannabidiol closely matched that of ω-conotoxin CVIE, a potent and selective Cav2.2 calcium channel blocker with proposed antinociceptive action suggesting that they too would block this channel. To verify this, Cav2.2 channels were heterologously expressed, recorded with whole-cell patch clamp and conolidine/cannabidiol was applied. Remarkably, conolidine and cannabidiol both inhibited Cav2.2, providing a glimpse into the MOA that could underlie their antinociceptive action. These data highlight the utility of cultured neuronal network-based workflows to efficiently identify MOA of drugs in a highly scalable assay.

## Introduction

In many cases, determining mechanisms of action (MOA) of naturally occurring compounds has proven difficult using standard pharmacological and physiological approaches. Natural compounds can act on multiple targets and standard assays may lack sufficient biological complexity to report the impact of multi-target molecules or to be sensitive to the highly non-linear mechanisms that affect neuronal excitability^1^. There is a clear need for workflows that can efficiently scale to the needs of central nervous system (CNS) drug discovery, assist in deconvoluting pharmacologic targets to help in understanding MOAs and efficiently predict *in vivo* efficacy and side effects or toxicity^2^. Advances in instrumentation, pattern recognition and cell and molecular biology have converged to create new opportunities for the development of innovative drug discovery workflows.

Cultures comprised of excitatory neurons, inhibitory neurons and glia self-assemble into spontaneously firing two-dimensional networks that share many functional and structural features with *in vivo* neuronal networks. A large number of neuronal network characteristics can be readily measured in these cultures using multi-electrode array (MEA) technology^3–5^. For example, network scale analysis can reveal the impact of drugs on emergent behaviour that would not be possible in single cell assays. To this end, cultured neuronal networks grown on MEAs coupled with pattern recognition techniques to build a database of drug-response functional profiles has aided the classification and identification of the MOA of novel chemical entities^6,7^.

Here, we show how this approach can be used to identify the pharmacological target of conolidine and cannabidiol, two chemically divergent naturally occurring antinociceptive agents. Conolidine is an alkaloid derived from the stem bark of the tropical flowering shrub *Tabernaemontana Divaricate* (Crepe Jasmine), which has been used in traditional Chinese, Thai and Ayurveda medicine for centuries for a range of ailments^8^. Tarselli *et al*.^9^ developed a synthetic route and went on to show a potent analgesic effect that compared favourably to opioids in chemically induced, inflammatory and acute tonic pain rodent models^9^ without many of the known liabilities of opioids. Despite considerable effort, the biological targets responsible for conolidine’s antinociceptive action could not be identified^9^. Cannabidiol is another naturally-occurring compound with complex MOAs, which is already used clinically in the management of pain^10,11^. Numerous targets have been implicated^12–14^, although the precise MOA is still under active investigation.

Our profiling and comparison of the actions of conolidine and cannabidiol with known pharmacological agents predicted a MOA shared with Ca_v_2.2 channel blocker, ω-conotoxin CVIE. *In vitro* analysis through whole cell patch clamping confirmed that, both conolidine and cannabidiol effectively blocked Ca_v_2.2 channels that are strongly implicated in pain^15,16^. This highlights the potential utility of MEA/cultured neuron assays in drug discovery pipelines.

## Methods

### Cell culture

#### Mouse cortical neurons

Experiments were carried out with multiwell MEAs from Multichannel Systems with each multiwell plate containing 24 wells. Each well contained 12 electrodes (diameter = 100 µM) spaced 700 µm apart. All MEAs were treated with polyethylenimine (PEI) (Sigma-Aldrich) 1 day before plating. Culture media was prepared with 89.3% Minimum Essential Medium (MEM, Merck KGaA), 0.9% 1 M HEPES (Sigma-Aldrich), 6 mg glucose (Merck KGaA) per 1 ml of MEM, 8.9% fetal bovine serum (Thermo Fisher Scientific) and 0.9% Penicillin/Streptomycin (P/S) solution (Sigma-Aldrich). Wells were coated with a 2% laminin (Sigma-Aldrich), 2–3 hours before plating and kept in the incubator. Laminin was removed immediately prior to plating.

Cortices were dissected from C57BL/6 0-2 post-natal mice pups and cultures were prepared based on the protocol described in^17^. Cortical pieces, pooled from multiple mice were subjected to dissociation using 0.25% Trypsin (Sigma-Aldrich), 0.032% DNase (Sigma-Aldrich) and trituration with a glass pipette.

375,000 cells were plated in 120 µl of media to cover the base of each well and after 1 hour, 300 µl of media was added. After 2 hours of further incubation, the initial media was removed and 500 µl of culture media containing Neurobasal-A solution was added. This media was prepared with Neurobasal-A medium (Thermo Fisher Scientific) supplemented with 1.9% B27 (Thermo Fisher Scientific), 0.95% GlutaMax (Thermo Fisher Scientific), 0.95% HEPES and 0.95% P/S. MEAs were covered with lids and kept in an incubator.

In order to control the proliferation of glia, 5 µM cytosine arabinoside (Merck KGaA) was added at 3 days *in vitro* (DIV) and was removed after 5 DIV with a 100% medium change. 50% culture medium changes were carried out twice a week. All animal experiments were approved by the Howard Florey Institute Animal Ethics Committee, and performed in accordance with the Prevention of Cruelty to Animals Act and the NHMRC Australian Code of Practice for the Care and Use of Animals for Scientific Purposes.

#### Mammalian cells, clones and transfection

Human embryonic kidney 293 T (HEK293T) cells were cultured according to procedures described previously^18^. HEK293T cells were transiently transfected using the calcium phosphate precipitation method^18^. The transfection mixture included human Ca_v_2.2 (N-type) channel (α_1B-1_; GenBank accession no.M94172.1; 2 μg; provided by Dr. D.T. Yue, The Johns Hopkins University School of Medicine, Baltimore, MD, USA), human α_2b_δ−1 (NM_000722, 2 μg; OriGene Technologies, Inc., Rockville, MD), human β_3a_ (NM_000725; 2 μg; OriGene), and enhanced green fluorescent protein (eGFP) reporter (0.5 μg; provided by J.W. Lynch, The University of Queensland, Brisbane, Australia).

HEK293T cells were seeded on 13 mm diameter glass coverslips (Menzel-Gläser, Thermo Fisher Scientific, Waltham, MA, USA) and incubated at 37 °C in 95% O_2_/5% CO_2_ for 6 h. Transfection medium was then replaced with culture medium consisting of Dulbecco’s Modified Eagle’s Medium (Thermo Fisher Scientific) supplemented with 10% (v/v) fetal bovine serum (Thermo Fisher Scientific), 50 IU/ml penicillin and 50 µg/ml streptomycin (Thermo Fisher scientific); cells were used within 3 days after transfection.

### MEA data acquisition

Data acquisition was carried out in 5-minute recordings at 20 kHz one day after changing the culture medium. Each culture grown in a MEA well was considered as one sample. MEAs were kept on the acquisition setup for 1 minute before recording, to allow cultures to regain normal activity levels after any mechanical stress caused by moving. During data acquisition, cultures were kept at 37 °C temperature and were kept in an enclosed chamber into which 95% O_2_/5% CO_2_ was continuously perfused. Data acquisition was carried out with the Multiwell MEA System and Multiwell_Screen software from Multi Channel Systems, Reutlingen, Germany.

### Selection and application of drugs in MEAs

Drugs were selected using two main criteria: (i) known mode of action and (ii) potential clinical analgesic properties. The known molecular target(s) of the drugs used are outlined in Table 1. Cultures at 14–21 DIV with more than 50% of channels having mean firing rates greater than 0.1 Hz were used for the application of drugs. A 100% media change was carried out one day prior. In each repeated sample of a drug, the baseline activity was recorded before perturbation and post-perturbation activity was recorded 10 minutes after drug application. All compounds were added in 5 µl aliquots to multiwell MEAs. Cultures were incubated in the intermediary period between recordings. Baclofen (G014 SIGMA), bicuculline (14340 SIGMA-ALDRICH), carbamazepine (C4024 SIGMA), diazepam (D-0899 SIGMA), Nickel Chloride Hydrate (364304 ALDRICH) and ZD7288 (Z3777 SIGMA) were sourced from Sigma-Aldrich, Castle Hill, NSW, Australia. LY341495 (ab120199) was sourced from Abcam, Melbourne, VIC, Australia and morphine from Glaxo Australia Pty Ltd. Conolidine was acquired from SYNthesis Med Chem, Parkville, Victoria, Australia, which was synthesized as described in^9^. Cannabidiol was sourced from Under the Tree BioPharmaceuticals Pty Ltd, Forte dei Marmi, Italy. CVIE was provided by Prof. Paul F. Alewood, Institute for Molecular Bioscience, University of Queensland, Brisbane, Australia. Stock solutions of compounds were initially prepared using solvents given in Table 1. These were further diluted in media and added to cultures so that the final concentrations in the bath were as shown in Table 1. Concentrations of drugs were selected by testing a range of concentrations around active concentrations reported in literature (Table 1) and selecting the concentration that achieved an approximated 50% reduction in mean firing rate. In the case of CVIE, morphine and bicuculline, high doses failed to produce significant reductions in mean firing and a concentration was selected based solely on literature analysis. Solvent concentration was kept at less than 0.1% of the culture medium.

**Table 1:** Molecular target(s), solvents and concentrations of drugs that were applied.

Drug	Target and Reference	Solvent	Stock Concentration	Concentration and number of samples
Conolidine	this study	DMSO	37.5 mM	30 µM (n = 18)
Cannabidiol	this study + Ca_v_3.1, 3.2, 3.3 (T-Type) channel blocker^40^, CB1, CB2 cannabinoid receptor antagonist^13^, µ and δ opioid receptor modulator^14^	DMSO	75 mM	10 µM (n = 18)
Baclofen	G protein-coupled gamma-aminobutyric acid (GABA) type B (GABA_B_) receptor agonist^42^	1 M NaOH	100 mM	1 µM^43^ (n = 18)
Bicuculline	GABA_A_ receptor antagonist^44^	DMSO	100 mM	20 µM^45^ (n = 18)
Carbamazepine	Na^+^ channel blocker^46^	DMSO	100 mM	10 µM^47^ (n = 18)
Morphine	µ-δ opioid receptor agonist^48^	H_2_O	50 mM	10 µM (n = 18)^49^
ω-conotoxin CVIE	Ca_v_2.2 (N-type) channel blocker^28^	H_2_O	—	100 nM (n = 18)^28^
Diazepam	GABA_A_ receptor positive allosteric modulator	DMSO	100 mM	1 µM^50^ (n = 18)
LY341495	Metabotropic glutamate (mGlu) receptor antagonist^51^	DMSO	100 mM	5 µM^51^ (n = 18)
Nickel (Ni^2+^)	Ca_v_3.1, Ca_v_3.2 and Ca_v_3.3 (T-type) channel blocker^52^	H_2_O	100 mM	200 µM^52^ (n = 18)
ZD7288	HCN channel blocker^53^	DMSO	100 mM	20 µM^54^ (n = 16)

### MEA data analysis

#### Feature extraction

Electrode voltage signals were high-pass filtered at 300 Hz following which spikes were detected with custom Matlab scripts based on precision timing spike detection^19^ with spikes being detected if the peak amplitude was greater than 6 times the standard deviation of noise. Noise levels were calculated in 20 s blocks.

Bursts in single channels (single–channel bursts) and network bursts were detected using an adaptive algorithm based on firing rates as described previously^20^. Bursts on single channels were detected as rapid successions of three or more spikes with inter-spike intervals lower than a threshold that adaptively changes based on firing rates. Network bursts were detected where more than 20% of single-channel bursts overlapped in time. Detected spikes, bursts and network bursts are illustrated in Supplementary Fig. 1.

Network characteristics were extracted in terms of firing and bursting parameters. Average amplitude of spikes and mean firing rates were calculated for each channel and these channel-wise means were averaged across channels again. Mean firing rates (MFR) were also calculated for bursting periods (MFRIn) and for non-bursting periods (MFROut). The ratio MFROut/MFRIn was calculated (MFRRatio) as well as the percentage of spikes inside bursts.

Burst features were calculated for both single-channel bursts and network bursts. Single-channel burst parameters include the number of spikes inside bursts and burst durations. Network burst parameter features consist of network burst durations, inter network burst intervals (INBI-time interval between the end of a network burst and the beginning of the next network burst), the amplitude of spikes in a network burst averaged over the network burst duration (avgNBAmp) and network burst jitter (Supplementary Fig. 1b). Jitter was defined as the onset time for channels that participated in the network burst. For burst parameters, the mean, coefficient of variation and range were calculated. The network burst rate and the average number of spikes in a network burst were also calculated. A full list of parameters and their definitions are included in Supplementary Table 1. For each parameter the percentage change from baseline was calculated.

#### Dimensionality reduction

As data acquisition methods and feature extraction methods advance, the analysis of multiparametric data becomes an absolute necessity. Multiparametric data analysis methods are common in fields such as bioinformatics where dimensionality reduction methods are used to reduce vast numbers of features into a few useful features^21,22^. Such analysis has also been used in the context of MEAs to a certain extent^4,23^. Therefore, we employ dimensionality reduction methods to reduce the complexity of multiparametric MEA data and compare responses of MEA cultures to different compounds.

For each sample, the percentage changes in parameters form a vector that describes its change in activity. Such vectors were calculated for all samples (1 sample = 1 MEA well) from all tested compounds. To compare a drug to a set of drugs with known MOA, feature values from all samples were first z-scored and PCA^24^ was performed on the set of feature vectors. Z-scoring ensures that all features have unit variance which prevents PCA from assigning higher importance to features with high variance. Principal components are orthogonal to each other, therefore using principal components overcomes the problem of extracted features being correlated to each other, which would otherwise bias similarity calculations between drugs. Each principal component describes a percentage of the variance of the data set and principal components are ordered according to this so that the first principal component describes the largest percentage of variance. Therefore, the first set of principal components that described 99% of the variance in data were extracted. Averages were calculated from the extracted principal component scores corresponding to the samples of each drug, resulting in an average vector per drug.

These average feature vectors were further reduced to two dimensions using Multi-Dimensional Scaling (MDS)^25^. MDS maps high-dimensional feature vectors into a lower dimensional space in a way that the dissimilarities between pairs of points are retained as much as possible. Dissimilarities were calculated as Euclidean distances between average feature vectors of drugs. The difference between dissimilarities in the original space and dissimilarities in the lower dimensional space were minimized by minimizing the ‘metric stress’ cost function which is defined as,

1 $$metric\,stress=\sqrt{\frac{{\sum }_{i\ne j}{({d}_{ij}-{d^{\prime} }_{ij})}^{2}}{{\sum }_{i\ne j}{d}_{ij}^{2}}}$$

where *d*_*ij*_ is the dissimilarity between sample i and j in the original n-dimensional space and ${d^{\prime} }_{ij}$ is the dissimilarity between the two samples in the two-dimensional space.

The similarity between a pair of drugs was calculated as the Euclidean distance between their positions in the final two-dimensional space.

#### Iris plots and analysis of effects on network parameters

In all cases, paired t-tests were performed to calculate statistically significant differences in the absence and presence of a drug using Matlab 2016a (The MathWorks, Inc., Natick, Massachusetts, United States). Radial heat maps, termed ‘iris plots’ were created in order to compare a given drug to a set of other drugs. These maps display multiple features of a particular drug as segments of a circle. Segments correspond to p-values resulting from statistical comparisons of the individual features in the absence and presence of a drug, and are represented by a log colour scale with red shades for increases in values and blue shades for decreases (Supplementary Fig. 2). P-values were adjusted for multiple comparisons using the Benjamini and Hochberg method^26^. Each iris plot serves as a signature for the responses evoked by a single drug and provides additional statistical insight that is separate from the comparison process involving PCA and MDS.

### Patch clamp electrophysiology

Step depolarization-activated Ba^2+^ currents (I_Ba_) through Ca_v_2.2 channels were recorded at room temperature (23–24 °C) in the whole-cell patch clamp configuration using an Axopatch 200B amplifier (Molecular Devices, Sunnyvale, CA) controlled by a Clampex 10/DigiData 1440 A acquisition system (Molecular Devices). Fire-polished borosilicate patch pipettes (GC150TF-7.5, Harvard Apparatus Ltd.) typically exhibited resistance values of 1.5–2.5 MΩ. For I_Ba_ measurements, the extracellular (bath) solution contained 10 mM BaCl_2_, 100 mM NaCl, 1 mM MgCl_2_, 5 mM CsCl, 30 mM TEA-Cl, 10 mM D-glucose, and 10 mM HEPES adjusted to a pH of 7.4 with TEA-OH, whereas the intracellular (pipette) solution contained 125 mM K-gluconate, 2 mM MgCl_2_, 5 mM EGTA, 5 mM NaCl, 4 mM MgATP, and 10 mM HEPES adjusted to a pH of 7.25 with CsOH. All compounds and reagents used to prepare extracellular and intracellular solutions were sourced from Sigma-Aldrich. Cells grown on coverslips were placed into a recording chamber with ~0.3 ml volume and superfused with bath solution at a constant rate of ~1 ml/min. Once whole-cell recording configuration was established, inward I_Ba_ currents were elicited from a holding potential of −80 mV using either step depolarizations from −40 to +50 mV, in 5-mV increments at 0.1 Hz stimulation frequency or single step depolarizations to +15 mV at 0.066 Hz. Experiments were only commenced when I_Ba_ amplitudes recorded during step depolarizations to +15 mV showed less than 5% change (usually decrease) within a 5-min pre-recording period. Membrane currents were filtered at 5 kHz and sampled at 20 kHz. I_Ba_ recordings, leak and capacitive currents were subtracted using a −P/4 pulse protocol. Conolidine, cannabidiol and CVIE were added to the extracellular solutions using stock solutions prepared as described in Table 1.

#### Data analysis

Data were analysed off-line using Clampfit 10 (Molecular Devices) and Origin 2017 (Microcal Software Inc., Northampton, MA). Current amplitudes obtained in the presence of a compound (I) were normalized to current amplitudes obtained under control conditions (I_max_). The fraction of conolidine or cannabidiol inhibition of I_Ba_ was determined as I/I_max_, whereas current recovery was defined as [(I_rec_ − I) / (I − I_max_)], where I_rec_ is the current amplitude after washout. Statistical analyses of patch-clamp data were performed using Sigma Plot 11.0 (Systat Software, Inc., San Jose, CA) or Graph-Pad Prism (GraphPad Software, La Jolla, CA).

## Results

### Effects of conolidine on network activity

The baseline properties of the 14–21 DIV neonatal cortical cultures were characterised. A total of 24 parameters were extracted and their ranges are shown in Supplementary Table 1. Network bursting across the majority of electrode channels was a consistent feature of these cultures and is a hallmark of network maturity (Supplementary Fig. 3)^23,27^. Another consistent feature of the network activity in these cultures was a prevalence of solitary spiking that occurred between network bursts. The consistency of network properties in these cultures provided a quantifiable framework on which to analyse the properties of drugs and test compounds. Average values of each network property are given in Supplementary Table 1.

**Figure 1:**
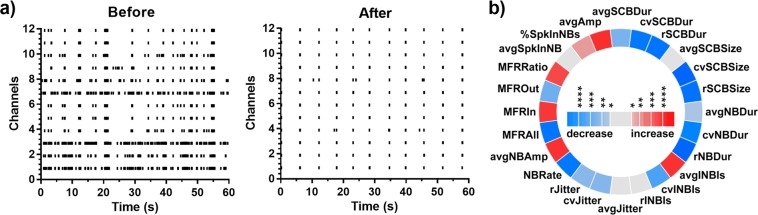
Changes in network behaviour evoked by 30 µM conolidine. (**a**) Representative raster plots of baseline activity of a culture before and after application of 30 µM conolidine. (**b**) Iris plot of conolidine. Each segment in the circle represents one feature. The colour scales represent the significance of p values (*p < 0.05, **p < 0.01, ***p < 0.001, ****p < 0.0001) resulting from paired t-tests (n = 18).

30 µM conolidine produced a stereotypical impact on network behaviour dominated by highly synchronous and periodic network bursting of consistent duration with complete absence of solitary spiking (Fig. 1a). This impact is reflected in quantifiable network features, including an increase in mean firing rates inside bursts relative to outside bursts (MFRRatio) and reduction in coefficients of variation in the duration of network bursts (cvNBDur) and the interval between network bursts (cvINBI) across 18 cultures. In addition to this, the overall firing rate (MFRAll) and network burst rate (NBRate) was also reduced by conolidine. A summary of the stereotypical effect of conolidine across cultures can be visualised in the circular p-value heat map or “iris plot” (Fig. 1b).

### Effects of cannabidiol on network activity

**Figure 2:**
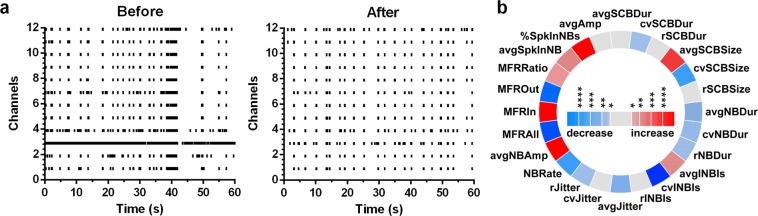
Changes in network behaviour evoked by 10 µM cannabidiol. (**a**) Representative raster plots of baseline activity of a culture and the activity after application of 10 µM conolidine (**b**) Iris plot of cannabidiol. Each segment in the circle represents one feature. The colour scales represent the significance of p values (*p < 0.05, **p < 0.01, ***p < 0.001, ****p < 0.0001) resulting from paired t-tests (n = 18).

10 µM cannabidiol impacted network activity similarly, but not identically, to conolidine (Fig. 2a). Cannabidiol also evoked synchronous, periodic network bursts with consistent durations and minimal solitary spiking. This is reflected as quantifiable network features in its iris plot (Fig. 2b) as an increase in mean firing rates inside bursts relative to outside bursts (MFRRatio) and reductions in the coefficients of variation in the duration of network bursts (cvNBDur) and the interval between network bursts (cvINBI). Similar to conolidine, cannabidiol also reduced overall firing and bursting (MFRAll, NBRate).

Even though cannabidiol increased the regularity of network bursting compared to baseline conditions, it could not achieve the level of regularity that conolidine evoked. This was evident when the coefficient of variances in network bursts intervals were compared between the two drugs (Student’s t-test, p = 0.0036).

### Generation of a drug response profile database

**Figure 3:**
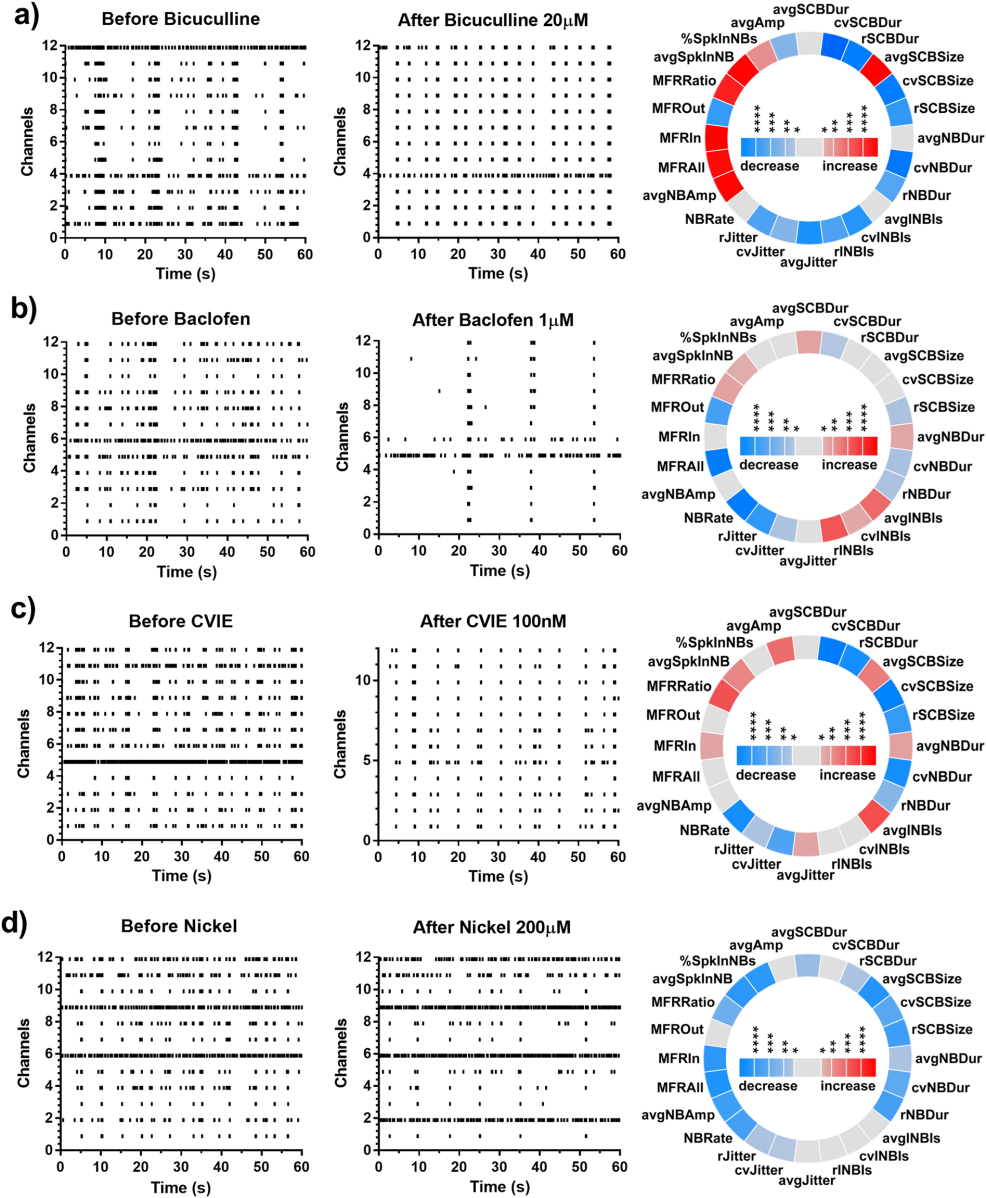
Changes in network behaviour evoked by drugs used for comparison. (**a**) 20 µM bicuculline, (**b**) 1 µM baclofen and (**c**) 200 µM Nickel (**d**) 100 nM CVIE. In each sub plot, representative raster plots of baseline activity of a culture, the activity after drug application and an iris plot showing the statistically significant changes in network features are shown. In the iris plot, each segment in the circle represents one feature. The colour scales represent the significance of p values (*p < 0.05, **p < 0.01, ***p < 0.001, ****p < 0.0001) resulting from paired t-tests.

A major goal of the present study was to identify the biological targets for conolidine and cannabidiol by comparison of drug effects in MEA cultures. We selected nine central nervous system (CNS) active compounds given in Table 1 based on the range of different targets that they engage. Although this is a somewhat limited selection it represents a broad pharmacological space including, voltage gated channels, ionotropic and metabotropic receptors. Response profiles for baclofen, bicuculline, Ni^2+^ and ω-conotoxin CVIE are shown in Fig. 3 and the remainder of the drugs in Table 1 are shown in the Supplementary Fig. 5.

Each drug caused distinct changes in the response profiles of the cultured neuronal networks (Fig. 3). For example, bicuculline, which is a GABA_A_ receptor antagonist, had a visually similar response profile to conolidine and cannabidiol, with synchronous periodic network bursting that was less frequent and with negligible solitary spiking (Fig. 3a). However, differences included increased firing rates and increased number of spikes in bursts. Baclofen, which is a GABA_B_ receptor agonist, decreased overall firing and bursting in cultures (Fig. 3b) similar to conolidine and cannabidiol but was unable to induce the same level of regularity. Ni^2+^, which is a T-type Ca^2+^ channel blocker, reduced firing, bursting and burst durations (Fig. 3c). Ω-conotoxin CVIE, a potent and selective Ca_v_2.2 channel blocker^28^, exhibited several similarities to conolidine and cannabidiol (Fig. 3d), including synchronous periodic network bursts which were fewer in number and negligible solitary spikes. The response profiles of the five remaining drugs also showed distinct properties with similarities and differences to conolidine and cannabidiol (Supplementary Fig. 5). Overall visual analysis of raster and iris plots was not sufficient to rank drugs that most closely shared response profiles with conolidine and cannabidiol. To address this, we employed multidimensional scaling and calculated the Euclidean distances between conolidine/cannabidiol and other drugs.

### Dimensionality reduction

**Figure 4:**
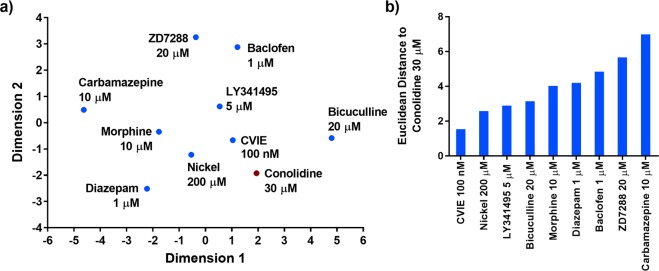
Similarity of conolidine to drugs with known mechanisms of action. (**a**) The position of conolidine 30 µM and compounds with known targets on the two-dimensional space created by multi-dimensional scaling. (**b**) The Euclidean distance between conolidine 30 µM and other drugs on the two-dimensional space.

Figure 4a shows the relationship of conolidine to other drugs on a two-dimensional plane created after multidimensional scaling showing strong separation of drug response profiles. The Euclidean distances between conolidine and each of the other drugs on the two-dimensional space are reported in Fig. 4b and provide an overall rank of similarity. By this measure, ω-conotoxin CVIE was most similar to conolidine in its response profile with a Euclidean distance of 1.5. Ni^2+^ was the second closest compound to conolidine with a distance of 2.6. Carbamazepine had the largest distance at 7.0. Bicuculline, though it had a visually similar activity profile, was positioned at a considerable distance from conolidine at 3.2.

**Figure 5:**
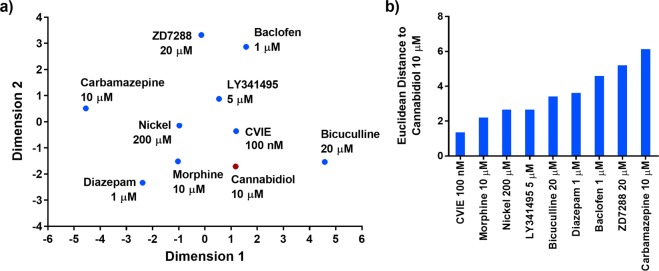
Similarity of cannabidiol to drugs with known mechanisms of action. (**a**) The position of cannabidiol 10 µM and compounds with known targets on the two-dimensional space created by multi-dimensional scaling. (**b**) The Euclidean distance between cannabidiol 10 µM and other drugs on the two-dimensional space.

Multidimensional scaling analysis was undertaken for cannabidiol (Fig. 5a) and interestingly quantification showed that its response profile was also most similar to ω-conotoxin CVIE with a Euclidean distance of 1.4 (Fig. 5b). Ni^2+^ and morphine were the second closest compounds to cannabidiol with distances of 2.2 and 2.7. Carbamazepine had the largest distance at 6.1. The distribution of individual sample points are included as Supplementary Figs 7 and 8.

### Ca_v_2.2 channel modulation by conolidine and cannabidiol

**Figure 6:**
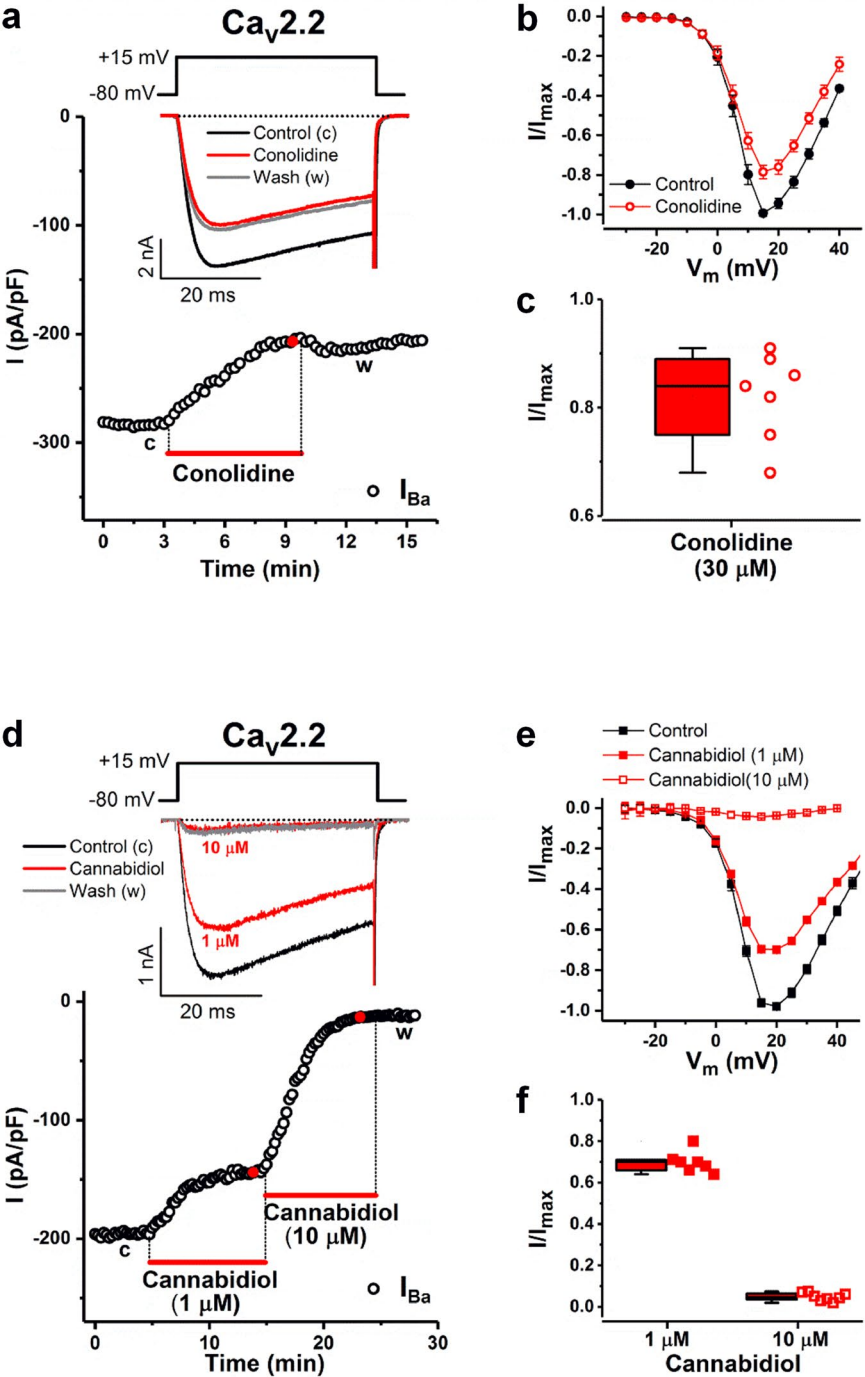
Conolidine and cannabidiol inhibition of currents through transiently expressed human Ca_v_2.2 channels. (**a**) Time course of I_Ba_ in the absence (control) and presence of 30 μM conolidine. Bar indicates conolidine application. I_Ba_ was evoked by 40-ms depolarizations to +15 mV, applied every 15 s from a holding potential of −80 mV (voltage inset). Representative inset I_Ba_ traces in the absence and presence of conolidine are shown at the times indicated by lowercase letters and the red filled circle, respectively. Horizontal dotted line represents zero-current level. (**b**) Mean current-voltage (I-V) relationships in the absence (control) and presence of conolidine (30 μM). (**c**) Summary of I_Ba_ inhibition by conolidine. The whisker box plot displays the median and the lower and upper quartiles; whiskers represent the minimum and maximum of the data (n = 7). (**d**) Time course of peak I_Ba_ in the absence and presence of 1 μM and 10 μM cannabidiol (lowercase letters and red filled circles, respectively). Data presentation and voltage protocol similar to that shown in a. (**e**) Mean I-V relationships in the absence (control) and presence of cannabidiol. (**f**) Summary of I_Ba_ inhibition by 1 μM and 10 μM cannabidiol (n = 7 and 8, respectively).

In order to test the target predictions from our MEA workflow we tested the impact of conolidine and cannabidiol on neuronal Ca_v_2.2 channels transiently expressed in HEK293T cells using whole-cell patch-clamp experiments (Fig. 6).

Conolidine, applied at the same concentration used in MEA analyses, inhibited depolarization-activated whole-cell I_Ba_ through Ca_v_2.2 channels (Fig. 6a). The effect developed slowly, occurred at physiologically relevant membrane potential (*V*_*m*_) values with no apparent change of the current-voltage (I–V) relationships, and showed poor reversibility (Fig. 6a,b). Quantification of multiple cells showed that 30 μM conolidine inhibited 17.9 ± 3% of the step depolarization-elicited inward peak I_Ba_ (n = 7) (Fig. 6c). Next, we examined whether cannabidiol could directly modulate Ca_v_2.2 channels. Figure 6d shows I_Ba_ through Ca_v_2.2 channels in the absence and presence of increasing concentrations (1 and 10 μM) of cannabidiol in the extracellular solution. Cannabidiol reduced peak I_Ba_ amplitude in a concentration and time-dependent manner (Fig. 6e,f), with10 μM resulting in full and irreversible inhibition. This data argues strongly that both conolidine and cannabidiol inhibition of Ca_v_2.2 channels significantly contributes to the modulation of neuronal network firing in cortical neuronal cultures and as a consequence a likely biological target for these compounds.

## Discussion

Conolidine and cannabidiol are two naturally occurring compounds that have antinociceptive properties. Here we identify Ca_v_2.2 channels as a common molecular target that may explain their shared action. We developed a workflow in which numerous firing parameters (recorded on MEA) are extracted from cultured neuronal networks and subjected to pattern recognition to identify similarity to known compounds. This workflow resulted in the nomination of Ca_v_2.2 channels as the likely targets that were verified using patch clamp analysis. This provides a potentially powerful method through which the MOA of unknown compounds can be efficiently determined.

Current workflows used for identifying the MOA of novel compounds generally involve an array of functional screening assays incorporating a myriad of isolated biological targets. These methods generally do not record function in the dynamic environment in which these targets usually reside. This is particularly important for ion channel targets that operate in very specialised and interactive temporal and spatial domains. The complexity of cultured networks provides a more realistic substrate on which to test CNS compounds. One difficulty of using this approach has been extracting and analysing unique signatures (or response profiles) for a given compound to create a similarity index. Existing methods that incorporate MEA recordings of cultured neuronal networks in their workflow rely largely on analysis of single parameters for identifying differences in drug action^29,30^. Because MEAs provide a richness of parameters, analyses that can incorporate the effects of a drug on all these parameters should be able to separate drugs more efficiently^5–7^. Unlike these earlier studies that used a training set of drugs to develop a classification scheme, here we use dimensionality reduction to develop an unsupervised method to compare drug response profiles.

Dimensionality reduction is used to reduce high-dimensional multiparametric data into a few informative dimensions. Reducing the number of dimensions also improves the visualizations of the spread of data. Classification, which is a supervised pattern recognition method, classifies a test input into a pre-defined set of output classes. In previous studies, drug similarities were identified by using drugs with known MOAs (training set) as classes and finding the probability of a test drug falling into each of these classes^6,7^. These classification methods require a larger number of samples per drug for training an accurate classification model and do not provide visualizations of the spread of drugs. Furthermore, unique characteristics in the response profile of a test drug, which did not exist in the training set of drugs, will not be captured in the model. In contrast, dimensionality reduction methods, such as multidimensional scaling, consider differences between all drugs and create a low-dimensional space that optimally retains these differences. Dimensionality reduction methods are therefore used widely in analysing biological data^21,22,31^. For unknown drugs that are considerably different from the know drugs that it is being compared to (ex: conolidine), this spatial spread indicates how different it is from the other drugs it is being compared to. Visualising drug placement also assists in identifying any additional drugs to add to the comparison.

To our knowledge dimensionality reduction has only been applied twice for comparing network profiles in MEA data^4,23^. In one study, PCA was used to identify neuronal network maturity levels while the other used PCA for separating drug classes but could only separate GABAA antagonists from other drugs. In the present study, we extend this use of dimensionality reduction by objectively quantifying the response profiles of a range of compounds with known actions to identify a molecular target for conolidine and cannabidiol. The failure of target panel-based screening to identify the MOA of conolidine^9^ highlights the potential advantages of approaches based on neuronal networks for target identification. Such approaches would not only assist in the identification of mechanisms or efficacy but could also provide a platform for compound safety and liability assessment.

Using PCA to reduce correlations between network parameters is a key feature of our workflow. When considering a multitude of parameters, it is inevitable that some of these parameters are correlated and giving the same importance (weight) to such correlated parameters diminishes the impact that other parameters have on the end result. An example of this is the multivariate analysis of variance (MANOVA) test that does not account for parameter correlation and cannot accurately determine similar drug responses (Supplementary Table 3). Other studies use feature selection methods to reduce the number of parameters^3,6^, with insufficient detail about the actual process. All feature selection methods do not necessarily select features (parameters) that have minimum correlations. Most methods would select features that best separates the training set of drugs. This would usually mean that the selected features consists of correlations and that these features would not capture unique characteristics of a new drug that has a completely different profile than those in the training set of drugs. Therefore, we use PCA instead of feature selection methods to reduce the dimensionality of our data.

Many studies use cortical cultures at maturity which produce rhythmic synchronised bursting^6,7^. However, using cultures at 14–21 DIV produced network activity that included both synchronised bursting as well as spiking in the intermediary periods, which provides greater variety in activity. Therefore, the changes captured when a drug is applied also have greater variety. For instance, with a mature cortical culture, its native activity would look very similar to conolidine’s and cannabidiol’s activity, making it difficult to capture the impact of the drug. Therefore, we believe that our selection of tissue type also contributes to the effectiveness of the workflow.

In our workflow, calculating average response profiles for each drug prior to dimensionality reduction also alleviates the problem of having small and unequal number of samples per drug, which most small-scale drug studies might face. Averaging all samples provides a more accurate representation of a drug, whereas individual samples may include considerable variations in the case of some drugs.

Our workflow is centred on building a database of network response profiles of compounds with known MOAs and comparing these with the response profiles of novel compounds with unknown action. Despite the small size of the drug database deployed, we were able to identify and experimentally confirm a target of conolidine and cannabidiol. This was aided by the careful selection of compounds to be included in the database that spanned a relatively wide range of pharmacological MOA. Developing a more comprehensive database of experimental compounds and approved drugs will significantly improve the utility of the proposed workflow. There are several advantages to this approach. 1. The database would only need to be generated once and it would naturally grow as it is utilised, 2. Cultured neuronal networks can readily scale to the analysis of thousands of compounds, 3. A larger database would permit the application of a broader array of pattern recognition methods. A further refinement would be the incorporation of ‘disease-state’ networks based on rodent or human stem cell-derived cultures harbouring mutations or modifications.

*In vitro* analysis revealed that conolidine and cannabidiol significantly blocked Ca_v_2.2 channels. In the peripheral and central nervous system, Ca_v_2.2 channels are located predominantly at presynaptic terminals^32^ and play essential roles in pain perception by modulating depolarization-induced calcium entry into neurons^33,34^. Compounds that affect presynaptic Ca_v_2.2 channel activity or the efficacy of calcium-dependent synaptic vesicle fusion are potential modulators of pain signalling. Importantly, ω-Conotoxin CVIE is known to inhibit nociceptive signalling and reverse allodynia in rodents^28^. Pathologically, dorsal horn Ca_v_2.2 channels expression is up-regulated in a rat sciatic nerve constriction model of neuropathic pain^35,36^, whereas Ca_v_2.2 knockout mice display reduced sensitivity to inflammatory and neuropathic pain^37–39^. Collectively, these data implicate Cav2.2 as a probable analgesic molecular target of conolidine and cannabidiol.

Our results suggest that conolidine and cannabidiol are likely to have other biological targets. For instance, multidimensional scaling analysis demonstrated a small Euclidean distance for the T-type calcium channel blocker Ni^2+^. Interestingly, cannabidiol is known to inhibit T-type channels^40^. T-type and Ca_v_2.2 channels have key roles in the generation and the propagation of spontaneous network driven bursts, respectively^41^. Therefore, the inhibition of network bursting observed here could be caused by both Ca_v_2.2 and/or Ca_v_3 (T-type) channel inhibition. Morphine also had a small Euclidean distance to cannabidiol and conolidine, and this may be a reflection of the fact that Ca_v_2.2 activity is efficiently modulated (inhibited) by G protein-coupled opioid receptor activation. This highlights that our approach may at times identify biological pathways implicated in the MOA rather than the target itself.

## Conclusion

Through our cortical culture/MEA-based workflow, we identified that the plant-derived compounds conolidine and cannabidiol act on Ca_v_2.2 (N-type) channels, which was corroborated through whole-cell patch-clamp assays. This common target may describe conolidine’s and cannabidiol’s shared antinociceptive properties. It is also probable that conolidine has multiple targets, similar to cannabidiol and other alkaloids with complex MOAs. Our study demonstrates that cortical culture/MEA analysis has the capacity to discover the pharmacodynamics of compounds with unknown MOAs. The development of human stem cell neuronal cultures used in conjunction with MEA technology promises to provide exciting new models on which to test compounds. Culture/MEA based workflows are therefore likely to become an integral part of the drug screening and development toolkit.

## Supplementary information

Supplementary Material

## Data Availability

The data that support the findings of this study are available from the corresponding author upon reasonable request.
