# Supplementary material for: Discovering the pharmacodynamics of conolidine and cannabidiol using a cultured neuronal network based workflow
Source: Sci Rep. 2019 Jan 15;9:121. doi: 10.1038/s41598-018-37138-w (PMC6333801; doi:10.1038/s41598-018-37138-w)
Supplement: Supplementary file 1 — Supplementary Material [file 41598_2018_37138_MOESM1_ESM.docx]

Discovering the pharmacodynamics of conolidine and cannabidiol using a cultured neuronal network based workflow

G D C Mendis^1^, G Berecki^2^, E Morrisroe^2^, S Pachernegg^2^, M Li^2^, M Varney^3^, P B Osborne^4^, C A Reid^2^, S Halgamuge^1,5^ and *S Petrou^2,4^

^1^Department of Mechanical Engineering, University of Melbourne, Parkville, VIC 3010, Australia

^2^Florey Institute of Neuroscience and Mental Health, University of Melbourne, Parkville, VIC 3010, Australia

^3^Neurolixis, Inc., Dana Point, CA 92629, USA

^4^Department of Anatomy and Neuroscience, University of Melbourne, Parkville, VIC 3010, Australia

^5^Research School of Engineering, College of Engineering and Computer Science, Australian

National University, Canberra ACT 0200, Australia

Corresponding Author: spetrou@unimelb.edu.au

# Supplementary Material

## Supplementary Table 1 - Definitions of extracted parameters and average values observed in baseline conditions

Average values were calculated using baseline values of all samples used in this study.

| Parameter | Mean ± Standard Error of Mean | Definition |
| --- | --- | --- |
| avgSCBDur | 87.99 ± 2.51 ms | Duration of single channel bursts averaged across all bursts in all bursting channels |
| cvSCBDur | 1.36 ± 0.03 | Coefficient of variation of durations of all single channel bursts in all bursting channels |
| rSCBDur | 1167.52 ± 49.63 ms | Range of durations of all single channel bursts in all bursting channels |
| avgSCBSize | 16.48 ± 0.53 | Number of spikes in single channel bursts averaged across all bursts in all bursting channels |
| cvSCBSize | 1.20 ± 0.03 | Coefficient of variation of the number of spikes in single channel bursts in all bursting channels |
| rSCBSize | 209.74 ± 10.80 | Range of the number of spikes in single channel bursts in all bursting channels |
| avgNBDur | 177.04 ± 4.26 ms | Network bursts durations averaged across all network bursts |
| cvNBDur | 0.89 ± 0.02 ms | Coefficient of variation of durations of network bursts |
| rNBDur | 877.38 ± 41.41 ms | Range of durations of network bursts |
| avgINBI | 3.12 ± 0.15 s | Inter network burst intervals averaged across all network bursts |
| cvINBI | 0.71 ± 0.01 | Coefficient of variation of inter network burst intervals |
| rINBI | 10.37 ± 0.51 s | Range of inter network burst intervals |
| avgJitter | 80.57 ± 1.87 ms | Jitter between single channel bursts participating in each network burst, averaged across all network bursts |
| cvJitter | 1.01 ± 0.03 | Coefficient of variation of the jitter between single channel bursts participating in a network burst |
| rJitter | 498.55 ± 27.93 ms | Range of the jitter between single channel bursts participating in a network burst |
| NBRate | 0.38 ± 0.02 Hz | Number of network bursts per second |
| avgNBAmp | 2.10 ± 0.08 µV | Sum of amplitudes of spikes included in a network burst divided by the network burst duration$\left( \frac{\sum Spike amplitudes}{Network burst duration} \right)$. Calculated per network burst and averaged across network burst |
| MFRAll | 6.91 ± 0.27 Hz | Mean firing rate $\left( MFR= \frac{Number of spikes}{Recording time} \right)$ calculated per channel and averaged across channels |
| MFRIn | 93.91 ± 2.99 Hz | Mean firing rate inside network bursts calculated per channel and averaged across channels |
| MFROut | 1.65 ± 0.17 Hz | Mean firing rate outside bursts calculated per channel and averaged across channels |
| MFRRatio | 1361.27 ± 140.35 | Ratio between MFRIn and MFROut $\left( MFR Ratio =\frac{MFRIn}{MFROut} \right)$ calculated per channel and averaged across channels |
| avgSpkInNBs | 190.53 ± 7.97 | Number of spikes included in each network burst averaged across network bursts |
| %SpkInNBs | 84.44 ± 1.02 % | Percentage of spikes included in network bursts calculated per channel and then averaged across channels |
| avgAmp | 29.88 ± 0.38 µV | Amplitude of spikes averaged across each channels and then averaged across all spiking channels |

## Supplementary Table 2: Means and standard errors of mean observed in the percentage change in features

| **Feature** | **Baclofen 1 µM** | | **Bicuculline 20 µM** | | **Cannabidiol 10 µM** | | **Carbamazepine 10 µM** | | **Conolidine 30 µM** | | **CVIE 100 nM** | |
| --- | --- | --- | --- | --- | --- | --- | --- | --- | --- | --- | --- | --- |
|  | **Mean** | **SEM** | **Mean** | **SEM** | **Mean** | **SEM** | **Mean** | **SEM** | **Mean** | **SEM** | **Mean** | **SEM** |
| avgSCBDur | 102.6 | 34.1 | 29.4 | 9.6 | 7.5 | 4.8 | 14.7 | 15.7 | -27.1 | 7.4 | 17.1 | 9.4 |
| cvSCBDur | -20.3 | 10.1 | -56.3 | 3.0 | -16.4 | 6.0 | 43.7 | 14.3 | -43.4 | 4.9 | -43.5 | 4.4 |
| rSCBDur | -22.1 | 11.4 | -70.9 | 3.2 | -14.5 | 10.1 | 74.1 | 29.8 | -64.4 | 3.4 | -44.7 | 6.8 |
| avgSCBSize | 74.0 | 27.7 | 117.9 | 19.9 | 24.3 | 6.0 | -45.4 | 5.3 | 7.7 | 8.7 | 30.9 | 9.5 |
| cvSCBSize | 0.5 | 13.1 | -46.7 | 4.0 | -22.7 | 5.0 | 7.6 | 4.5 | -56.3 | 3.6 | -42.9 | 4.2 |
| rSCBSize | -22.2 | 11.3 | -49.8 | 8.9 | -7.8 | 11.9 | -30.7 | 7.0 | -73.8 | 2.9 | -47.9 | 6.3 |
| avgNBDur | 85.1 | 30.6 | 20.6 | 7.4 | -14.8 | 4.0 | 81.1 | 73.9 | -18.6 | 7.8 | 25.2 | 8.6 |
| cvNBDur | -28.0 | 10.6 | -64.0 | 4.7 | -19.8 | 6.5 | 39.9 | 6.7 | -47.0 | 5.7 | -42.1 | 5.1 |
| rNBDur | -31.0 | 10.8 | -52.7 | 9.7 | -25.5 | 8.7 | 122.5 | 72.8 | -63.8 | 3.4 | -31.3 | 12.1 |
| avgINBIs | 986.5 | 226.2 | 17.1 | 11.6 | 33.2 | 8.4 | 7.9 | 30.5 | 139.3 | 24.4 | 54.8 | 11.8 |
| cvINBIs | 37.9 | 17.9 | -32.5 | 5.3 | -39.6 | 2.6 | 64.5 | 16.3 | -37.5 | 8.3 | -8.7 | 6.4 |
| rINBIs | 569.1 | 146.3 | -33.5 | 6.5 | -9.5 | 10.2 | 129.0 | 66.5 | 12.6 | 17.0 | 12.2 | 12.4 |
| avgJitter | -6.5 | 14.1 | -50.7 | 6.9 | -23.3 | 6.2 | 150.8 | 128.9 | -9.8 | 9.2 | 27.6 | 8.0 |
| cvJitter | -27.1 | 11.5 | -38.5 | 10.9 | -13.4 | 6.8 | 35.5 | 6.2 | -26.9 | 7.7 | -25.4 | 4.0 |
| rJitter | -57.5 | 8.4 | -67.6 | 8.8 | -36.3 | 7.6 | 138.4 | 86.5 | -41.5 | 8.1 | -17.3 | 9.4 |
| NBRate | -85.9 | 7.0 | -2.4 | 8.0 | -18.2 | 4.8 | 27.4 | 15.8 | -49.8 | 4.5 | -29.3 | 4.7 |
| avgNBAmp | -2.7 | 20.1 | 130.3 | 18.9 | 60.0 | 11.3 | -69.1 | 4.4 | 54.9 | 8.3 | -0.8 | 4.7 |
| MFRAll | -75.6 | 5.3 | 126.3 | 26.1 | -45.1 | 2.6 | -43.3 | 6.2 | -52.4 | 3.9 | -6.3 | 5.6 |
| MFRIn | 8.6 | 15.2 | 136.6 | 22.9 | 42.5 | 8.7 | -68.9 | 3.5 | 49.2 | 10.8 | 12.0 | 5.2 |
| MFROut | -56.9 | 8.1 | -40.8 | 24.5 | -79.6 | 2.1 | 43.0 | 25.2 | -75.2 | 5.0 | -20.7 | 11.2 |
| MFRRatio | 1162 | 338.5 | 1749 | 514 | 938 | 198 | -90.5 | 2.2 | 1686 | 357 | 712 | 258 |
| avgSpkInNB | 93.5 | 28.3 | 204.9 | 40.3 | 18.4 | 5.9 | -52.5 | 18.1 | 17.0 | 11.7 | 40.7 | 11.9 |
| %SpkInNBs | -15.5 | 9.4 | 33.5 | 24.1 | 40.0 | 4.8 | -21.8 | 6.3 | 7.4 | 2.8 | 3.0 | 2.4 |
| avgAmp | -4.7 | 2.4 | -5.8 | 1.7 | 5.2 | 3.7 | -15.5 | 2.3 | 12.6 | 2.3 | 4.4 | 1.3 |

| **Feature** | **Diazepam**  **1 µM** | | **LY341495**  **5 µM** | | **Morphine**  **10 µM** | | **Nickel**  **200 µM** | | **ZD7288**  **20 µM** | |
| --- | --- | --- | --- | --- | --- | --- | --- | --- | --- | --- |
|  | **Mean** | **SEM** | **Mean** | **SEM** | **Mean** | **SEM** | **Mean** | **SEM** | **Mean** | **SEM** |
| avgSCBDur | -43.9 | 2.2 | 19.9 | 11.2 | -0.8 | 4.4 | -21.6 | 6.0 | 159.6 | 27.8 |
| cvSCBDur | 11.4 | 5.6 | -29.0 | 4.9 | 0.9 | 4.4 | -11.6 | 8.3 | -24.3 | 5.3 |
| rSCBDur | -54.5 | 5.9 | -39.8 | 8.7 | 8.4 | 20.1 | -24.8 | 18.6 | -5.5 | 9.7 |
| avgSCBSize | -55.9 | 1.8 | 14.5 | 7.7 | 0.7 | 4.7 | -36.0 | 3.9 | 61.9 | 11.9 |
| cvSCBSize | -0.4 | 6.5 | -25.5 | 5.6 | 6.5 | 5.0 | -29.1 | 4.9 | 8.1 | 10.4 |
| rSCBSize | -55.2 | 7.6 | -43.3 | 9.9 | 4.2 | 8.6 | -62.3 | 5.3 | 19.6 | 39.2 |
| avgNBDur | -40.2 | 2.7 | 19.5 | 13.9 | -6.1 | 3.1 | -16.9 | 6.3 | 232.4 | 42.9 |
| cvNBDur | -1.6 | 5.3 | -28.7 | 6.4 | 5.3 | 5.9 | -29.1 | 5.4 | -38.9 | 6.4 |
| rNBDur | -44.4 | 6.2 | -32.6 | 13.2 | 1.7 | 7.5 | -48.4 | 6.3 | 17.2 | 16.2 |
| avgINBIs | -39.7 | 5.4 | 248.8 | 32.2 | -16.9 | 5.2 | 65.7 | 18.4 | 498.8 | 75.1 |
| cvINBIs | -28.0 | 4.7 | 40.1 | 11.1 | 9.0 | 4.5 | 2.9 | 12.0 | -15.6 | 9.1 |
| rINBIs | -43.1 | 5.6 | 292.9 | 76.0 | -11.1 | 5.6 | 102.5 | 46.3 | 194.4 | 41.5 |
| avgJitter | -25.3 | 4.2 | 12.1 | 13.8 | 2.4 | 4.1 | -1.9 | 8.6 | 225.4 | 56.5 |
| cvJitter | -1.6 | 6.1 | -25.3 | 5.7 | 2.5 | 4.5 | -15.0 | 5.6 | -33.2 | 5.4 |
| rJitter | -32.5 | 8.5 | -37.6 | 9.9 | 8.5 | 9.1 | -28.9 | 7.7 | 18.6 | 16.8 |
| NBRate | 84.9 | 12.9 | -60.2 | 10.1 | 29.2 | 8.5 | -25.9 | 8.3 | -76.1 | 3.9 |
| avgNBAmp | -50.1 | 3.3 | 2.1 | 7.4 | -3.9 | 7.7 | -30.4 | 6.6 | -22.0 | 13.5 |
| MFRAll | -33.5 | 3.5 | -57.9 | 3.8 | 11.0 | 4.8 | -49.3 | 3.8 | -50.7 | 5.9 |
| MFRIn | -44.7 | 3.4 | 10.8 | 8.1 | -0.1 | 5.5 | -25.8 | 6.3 | -21.8 | 4.3 |
| MFROut | 24.1 | 7.8 | 3.0 | 41.8 | -1.2 | 3.7 | 15.0 | 11.0 | -62.0 | 6.9 |
| MFRRatio | -59.8 | 11.8 | 2663.6 | 1219.2 | 7.6 | 26.0 | -30.8 | 13.2 | 417.0 | 98.9 |
| avgSpkInNB | -67.3 | 2.1 | 22.0 | 8.7 | -7.1 | 6.1 | -43.5 | 4.9 | 125.3 | 37.3 |
| %SpkInNBs | -9.1 | 1.2 | -3.4 | 2.9 | 1.4 | 0.5 | -12.9 | 3.3 | -1.6 | 4.9 |
| avgAmp | -13.0 | 0.9 | -1.8 | 1.3 | 0.5 | 1.3 | -2.2 | 1.2 | -0.7 | 3.4 |

## Supplementary Table 3: p-values resulting from paired MANOVA tests between Conolidine / Cannabidiol and each of the other drugs

Multivariate analysis of variance (MANOVA) tests were carried out using the Hotelling-Lawley method in RStudio (RStudio Team 2016, RStudio: Integrated Development for R. RStudio Inc., Boston, MA). According to p-values resulting from MANOVA, Baclofen would be the closest drug to both Conolidine and Cannabidiol. This shows that simple statistical tests such MANOVA cannot accurately determine similar drug responses, mainly due to some measured parameters being correlated to each other.

|  | **Conolidine** | **Cannabidiol** |
| --- | --- | --- |
| Baclofen | 0.04365 | 0.001422 |
| Bicuculline | 0.00000003471 | 0.000003259 |
| Carbamazepine | 0.000005783 | 0.000002703 |
| CVIE | 0.0004207 | 0.0001061 |
| Diazepam | 0.000006958 | 0.00002599 |
| LY341495 | 0.001035 | 0.00004353 |
| Morphine | 0.000002787 | 0.000012 |
| Nickel | 0.0001154 | 0.0001456 |
| ZD7288 | 0.001152 | 0.00000246 |

## Supplementary Figure 1: Feature extraction in multielectrode array data


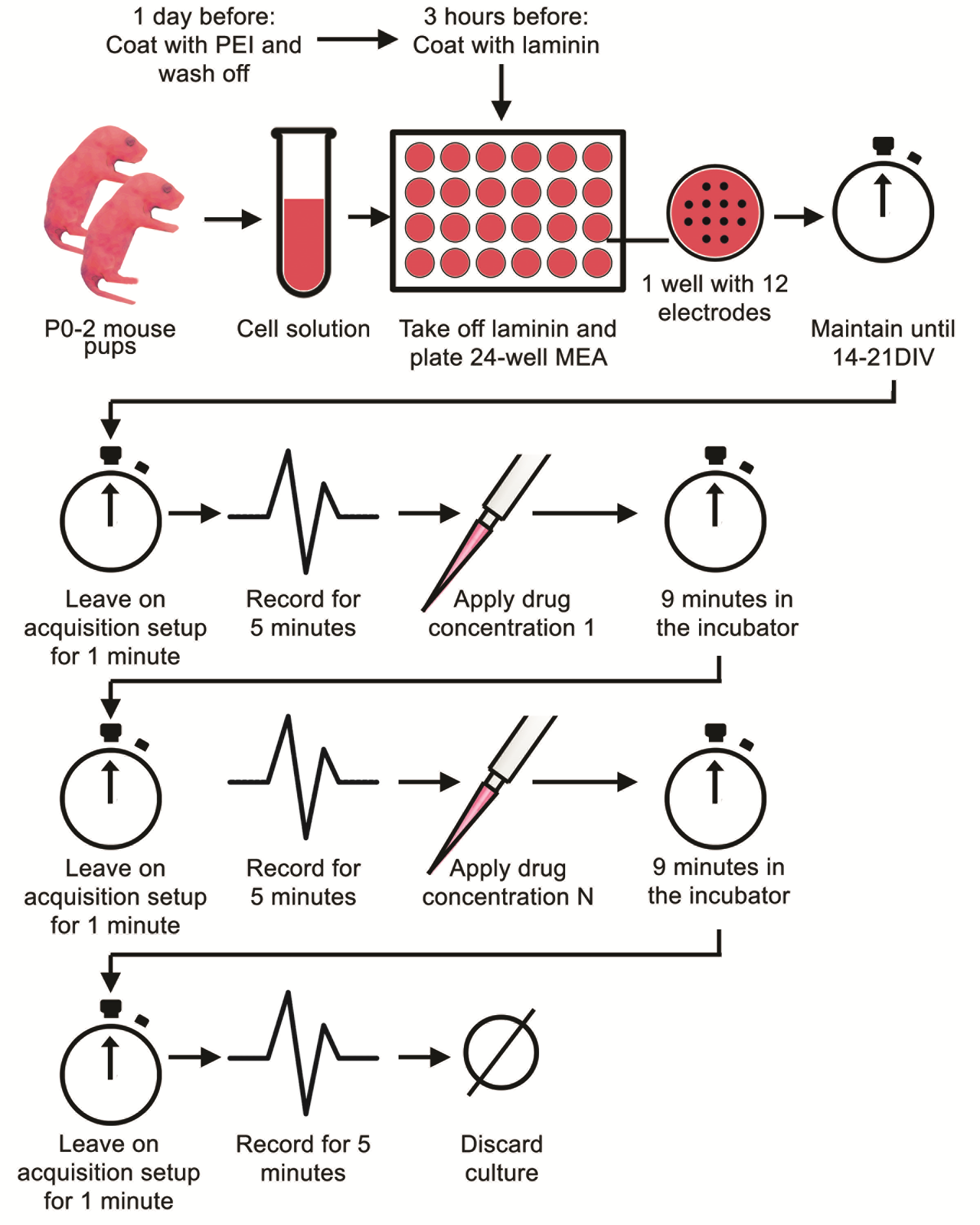


Supplementary Figure 1: The experimental workflow of testing a single drug on multielectrode arrays

## Supplementary Figure 2: Feature extraction in multielectrode array data





Supplementary Figure 2: (a) 500 ms recording of 12 channels during a cluster of bursts. Noise is represented in grey while action potentials are shown in red. Action potentials are detected when they cross a threshold set as a multiple of the standard deviation of noise. (b) Dynamics of a neuronal network that can be visualized through raster plots. Each vertical line on a channel represents an action potential (spike). A succession of spikes in close temporal proximity constitutes to a burst, as shown by the group of spikes enclosed by a black rectangle. Simultaneous bursting across multiple channels, as shown in red, corresponds to a network burst (NB). The interval of time from the end of a network burst to the beginning of the next network burst is termed as an Inter-Network Burst Interval (INBI). The time interval between the start of the first single channel burst and the start of the last single-channel burst in a network burst is termed as jitter.

## Supplementary Figure 3: Iris Plot


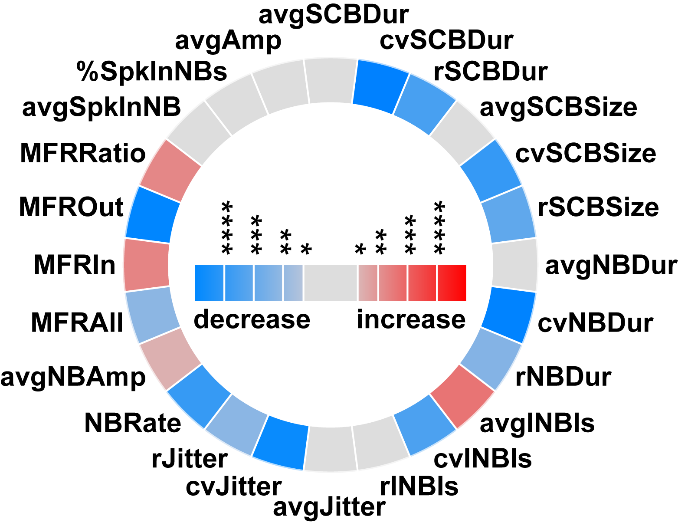


Supplementary Figure 3: Iris plot. Each segment in the circle represents one feature. The colour scales represent the significance of p values (* - p < 0.05, ** - p < 0.01, *** - p < 0.001, **** - p < 0.0001) resulting from paired t-tests (corrected for multiple comparisons) between before and after conditions of a given drug. The red colour scale represents increases and the blue colour scale represents decreases from baseline conditions.

## Supplementary Figure 4: Raster plots of baseline activity





Supplementary Figure 4: Raster plots of baseline activity of three cultures, which show network bursting and non-synchronous spikes that occurred between network bursts. Black ticks in each channel represent spike timestamps while red ticks at the bottom represent detected network bursts.

## Supplementary Figure 5: Changes in network behavior evoked by 0.1% DMSO





Supplementary Figure 5: Changes in network behaviour evoked by 0.1% DMSO. (a) Representative raster plots of baseline activity of a culture and the activity after application of 0.1% DMSO. (b) Iris plot of conolidine. Each segment in the circle represents one feature. The colour scales represent the significance of p values (* - p < 0.05, ** - p < 0.01, *** - p < 0.001, **** - p < 0.0001) resulting from paired t-tests (n=12) corrected for multiple comparisons.

## Supplementary Figure 6: Changes in network behavior evoked by Morphine, LY341495, ZD7288, Diazepam and Carbamazepine


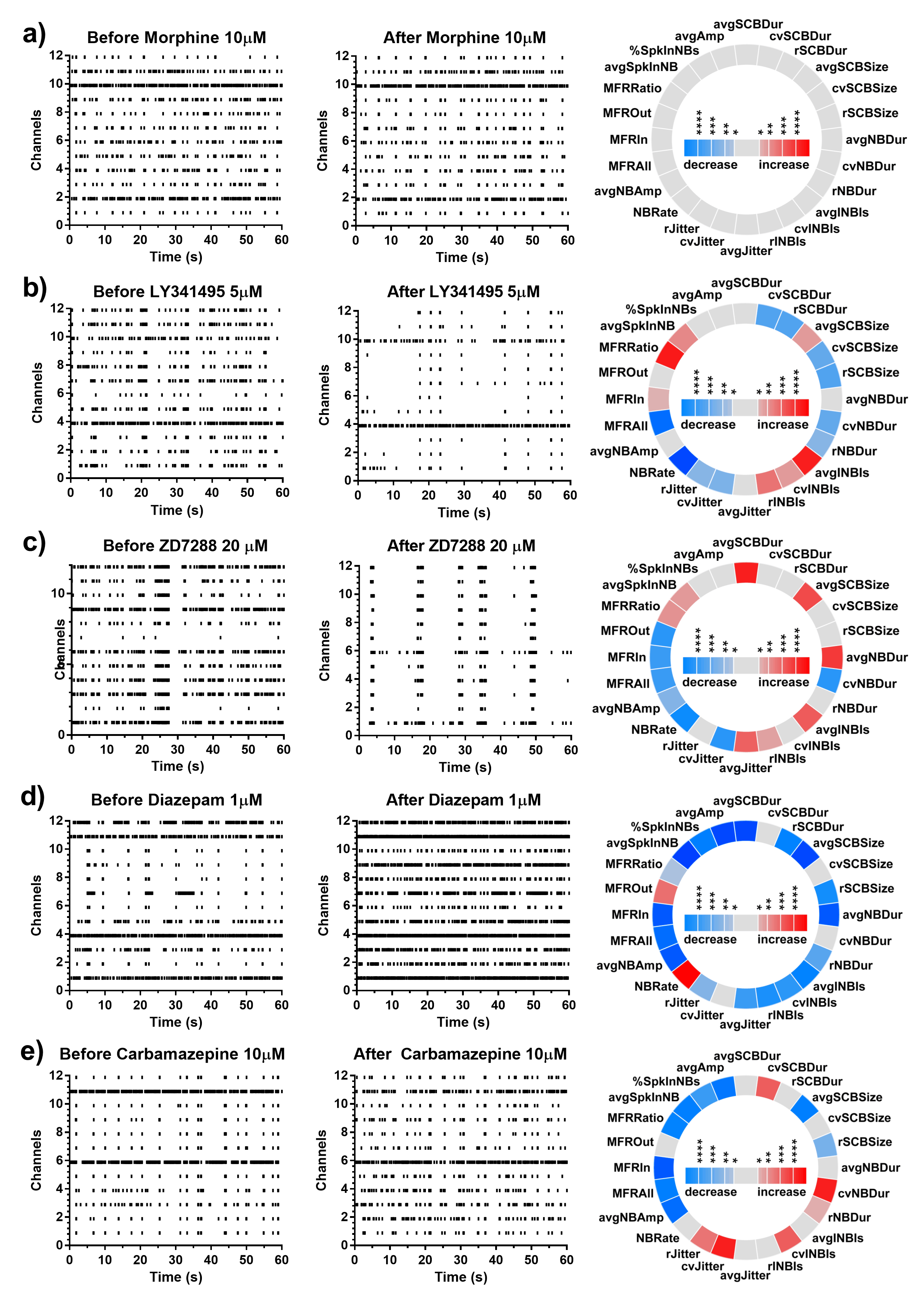


Supplementary Figure 6: Changes in network behaviour evoked by (a) 10 µM Morphine, (b) 5 µM LY341495, (c) 20 µM ZD7288, (d) 1 µM Diazepam and (e) 10 µM Carbamazepine. In each sub plot, representative raster plots of baseline activity of a culture, the activity after drug application and an iris plot showing the statistically significant changes in network features are shown. In the iris plot, each segment in the circle represents one feature. The colour scales represent the significance of p values (* - p < 0.05, ** - p < 0.01, *** - p < 0.001, **** - p < 0.0001) resulting from paired t-tests corrected for multiple comparisons.

## Supplementary Figure 7: Distribution of individual samples after Principal Component Analysis and Multi-Dimensional Scaling for Conolidine and other drugs


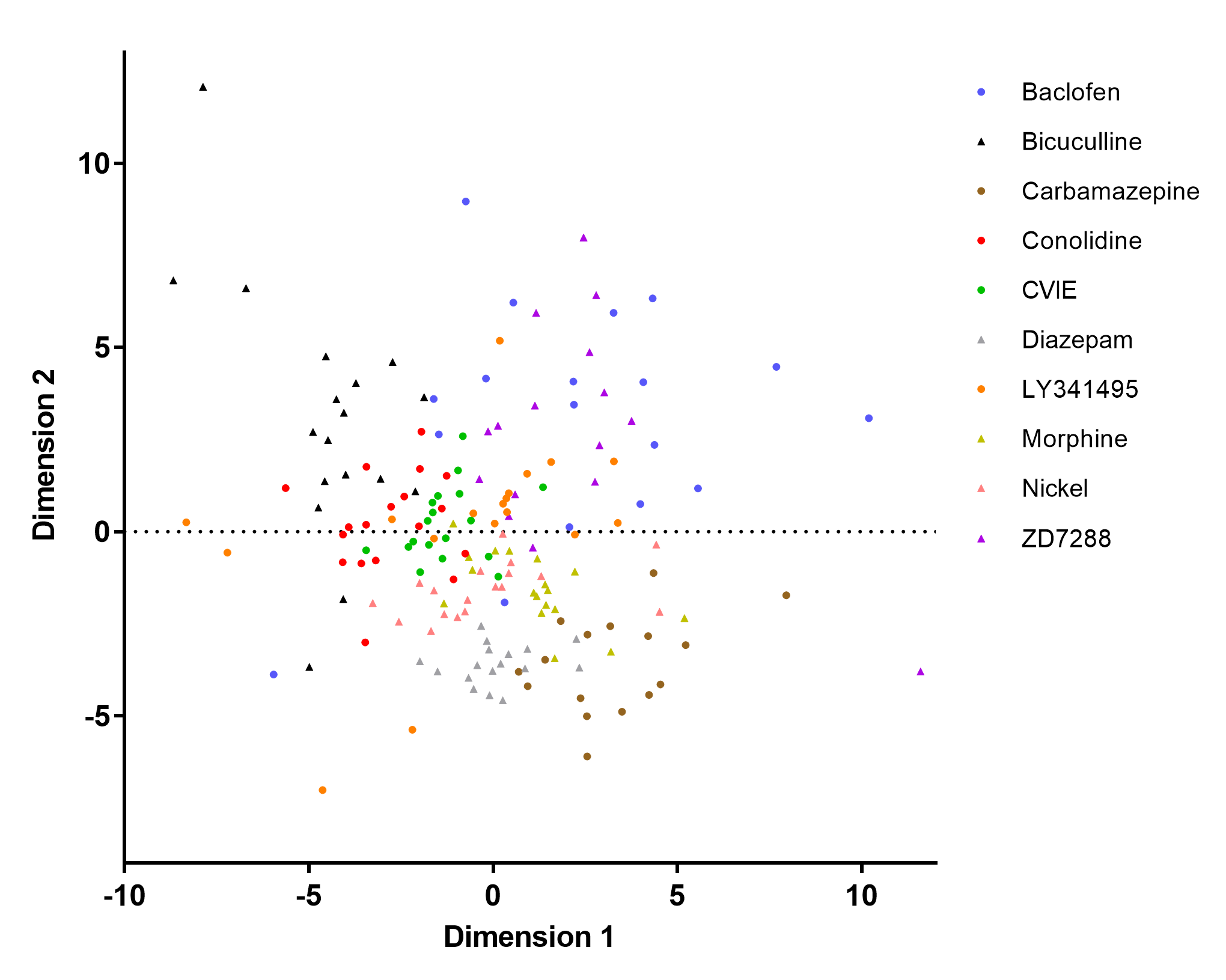


## Supplementary Figure 8: Distribution of individual samples after Principal Component Analysis and Multi-Dimensional Scaling for Cannabidiol and other drugs


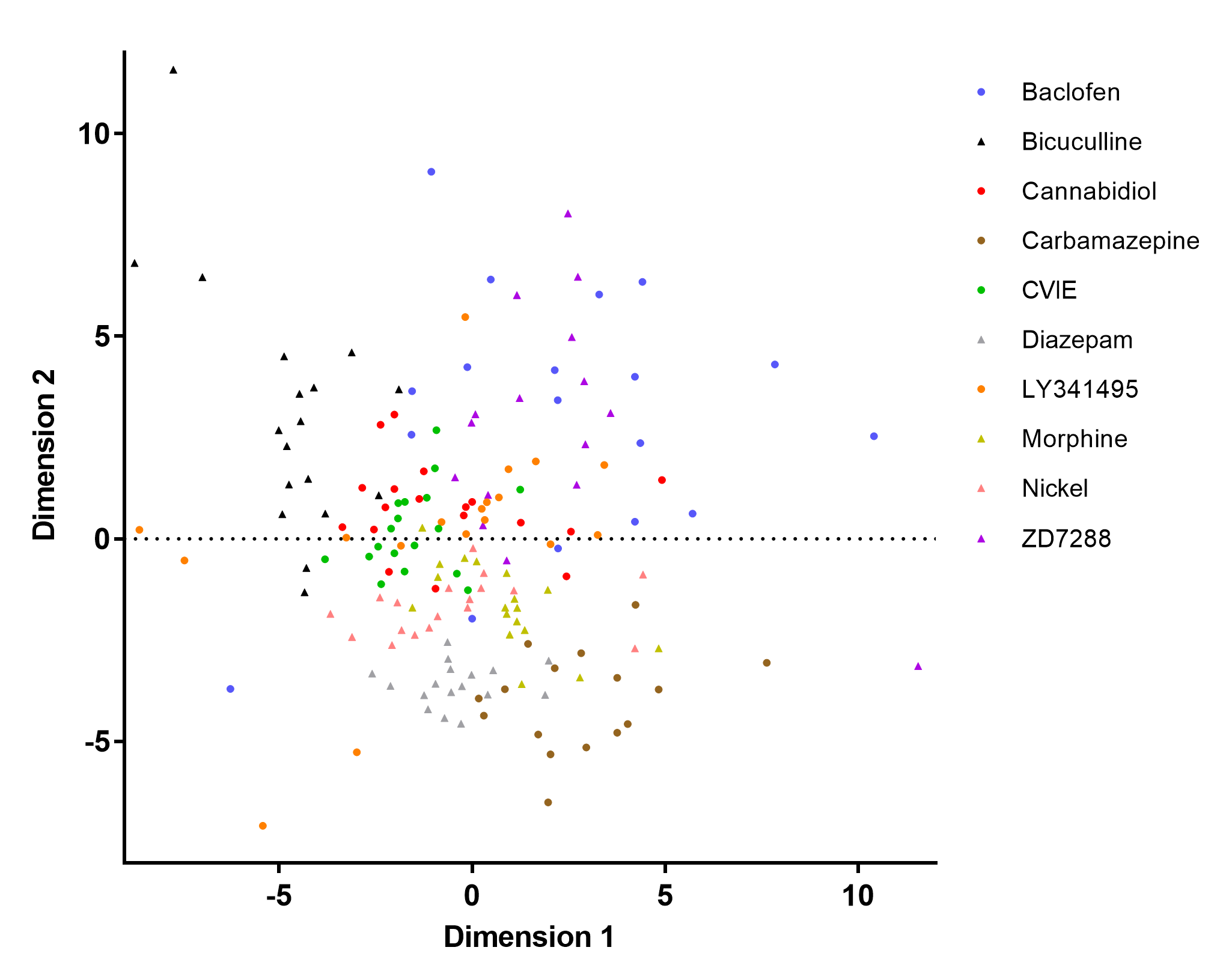


* Scales have been set for optimum visibility of the center of the cluster of points without excluding too many outliers
